# Supplementary material for: Cross-Sectional and Longitudinal Associations between Skin Autofluorescence and Tubular Injury Defined by Urinary Excretion of Liver-Type Fatty Acid-Binding Protein in People with Type 2 Diabetes
Source: Biomedicines. 2023 Nov 10;11(11):3020. doi: 10.3390/biomedicines11113020 (PMC10669246; doi:10.3390/biomedicines11113020)
Supplement: Supplementary file 1 [file biomedicines-11-03020-s001.zip › biomedicines-2676721-supplementary.pdf]

Table S1. Multiple linear regression analysis for determinants (including eGFR but not Cr) of DKD biomarkers

Variables	uACR			Log-transformed uACR			uL-FABPCR			Log-transformed uL-FABPCR		
	t value	VIF	*p* value	*t* value	VIF	*p* value	*t* value	VIF	*p* value	*t* value	VIF	*p* value
Age	-2.327	1.839	0.021	0.261	1.839	0.794	-0.399	1.839	0.690	1.701	1.839	0.090
Male	-0.346	1.383	0.730	-0.183	1.383	0.855	-0.882	1.383	0.379	-1.700	1.383	0.090
BMI	1.663	1.556	0.097	3.249	1.556	0.001	2.087	1.556	0.038	2.171	1.556	0.031
Current smoking	0.847	1.222	0.398	1.867	1.222	0.063	1.476	1.222	0.141	1.792	1.222	0.074
SBP	1.751	1.304	0.081	1.521	1.304	0.129	0.671	1.304	0.502	0.244	1.304	0.807
TG	2.773	1.473	0.006	1.205	1.473	0.229	0.639	1.473	0.523	-0.531	1.473	0.596
HDL-C	-0.466	1.374	0.642	-1.052	1.374	0.294	-0.317	1.374	0.751	-0.630	1.374	0.529
LDL-C	3.432	1.249	0.001	1.853	1.249	0.065	2.696	1.249	0.007	1.033	1.249	0.303
Casual PG	2.646	1.652	0.009	0.987	1.652	0.325	1.065	1.652	0.288	0.850	1.652	0.396
HbA1c	-0.811	1.684	0.418	0.384	1.684	0.701	-0.032	1.684	0.974	1.575	1.684	0.116
UA	-1.689	1.484	0.092	-1,604	1.484	0.110	-1.493	1.484	0.136	-1.607	1.484	0.109
eGFR	-4.311	1.460	<0.001	-3.357	1.460	0.001	-3.876	1.460	<0.001	-2.894	1.460	0.004
Hypertension	0.942	1.322	0.347	3.244	1.322	0.001	1.395	1.322	0.164	2.276	1.322	0.024
Duration of T2D	3.427	1.260	0.001	3.420	1.260	0.001	3.595	1.260	<0.001	2.842	1.260	0.005
Dyslipidemia	-0.108	1.148	0.914	0.123	1.148	0.903	-0.547	1.148	0.585	0.259	1.148	0.796
SAF	1.825	1.290	0.069	1.817	1.290	0.070	2.481	1.290	0.014	2.196	1.290	0.029

Table S2. Multiple linear regression analysis including identified confounding factors (including eGFR but not Cr) and medications used for determinants of uL-FABPCR

Variables	Model 1						Model 2					
	uL-FABPCR			Log-transformed uL-FABPCR			uL-FABPCR			Log-transformed uL-FABPCR		
	t value	VIF	*p* value	*t* value	VIF	*p* value	*t* value	VIF	*p* value	*t* value	VIF	*p* value
Age	-	-	-	-	-	-	-	-	-	-	-	-
Male	-	-	-	-	-	-	-	-	-	-	-	-
BMI	2.630	1.181	0.009	1.715	1.198	0.087	2.435	1.226	0.015	1.091	1.277	0.2763
Current smoking	-	-	-	-	-	-	-	-	-	-	-	-
LDL-C	3.230	1.256	0.001	-	-	-	2.885	1.070	0.004	-	-	-
UA	-	-	-	-	-	-	-	-	-	-	-	-
eGFR	-3.517	1.102	<0.001	-3.251	1.101	0.001	-3.786	1.116	<0.001	-3.068	1.120	0.002
Hypertension	-	-	-	1.794	1.829	0.074	-	-	-	2.874	1.125	0.004
Duration of T2D	3.523	1.201	<0.001	3.120	1.188	0.002	3.380	1.557	<0.001	1.549	1.560	0.122
SAF	3.085	1.184	0.002	2.926	1.194	0.004	3.246	1.220	0.001	2.643	1.253	0.009
ARB or ACEi	0.132	1.338	0.895	-0.802	1.659	0.423	-	-	-	-	-	-
CCB	3.391	1.378	<0.001	2.242	1.498	0.026	-	-	-	-	-	-
β blocker	-2.144	1.070	0.033	-2.121	1.075	0.035	-	-	-	-	-	-
MR blocker	-1.564	1.067	0.119	-1.770	1.066	0.078	-	-	-	-	-	-
Statin	-0.479	1.225	0.633	-0.768	1.087	0.443	-	-	-	-	-	-
Ezetimibe	0.068	1.077	0.946	1.090	1.066	0.277	-	-	-	-	-	-

Other hypolipidemic drugs	-0.636	1.047	0.525	0.226	1.046	0.822	-	-	-	-	-	-
Antiplatelets	0.474	1.140	0.636	0.441	1.143	0.660	-	-	-	-	-	-
SU or Glinide	-	-	-	-	-	-	-0.871	1.274	0.384	0.667	1.275	0.505
Metformin	-	-	-	-	-	-	-0.405	1.268	0.686	-1.564	1.268	0.119
DPP-4i	-	-	-	-	-	-	-1.208	1.501	0.228	0.266	1.498	0.790
SGLT2i	-	-	-	-	-	-	0.808	1.198	0.420	3.161	1.199	0.002
αGI	-	-	-	-	-	-	-0.098	1.218	0.922	0.438	1.213	0.662
Pioglitazone	-	-	-	-	-	-	1.222	1.082	0.222	0.233	1.082	0.816
Insulin	-	-	-	-	-	-	-0.015	1.240	0.988	1.206	1.257	0.229
GLP-1RA	-	-	-	-	-	-	0.889	1.505	0.375	0.540	1.486	0.590

Table S3. Clinical characteristics of subjects at baseline in the longitudinal study

	Total	Males	Females	p value (Males vs Females)
Number of subjects	220	128	92	
Age (years)	71 (62, 75)	71 (63, 77)	69 (62, 72)	0.250
BMI (kg/m^2)	24.3 (22.1, 26.9)	24.3 (22.1, 26.1)	24.4 (22.0, 28.6)	0.250
SBP (mmHg)	132.5 (123.0, 143.0)	131.0 (123.0, 142.0)	135.0 (125.0, 145.3)	0.336

TG (mmol/L)	1.3 (0.9, 1.7)	1.4 (1.0, 1.7)	1.2 (0.8, 1.6)	0.561
HDL-C (mmol/L)	1.3 (1.1, 1.6)	1.3 (1.1, 1.5)	1.5 (1.2, 1.7)	<0.001
LDL-C (mmol/L)	2.5 (2.0, 3.0)	2.5 (2.0, 3.0)	2.5 (2.1, 3.0)	0.750
Casual PG (mmol/L)	7.5 (6.2, 8.8)	7.6 (6.7, 10.2)	6.7 (6.1, 7.8)	<0.001
HbA1c (%)	6.7 (6.4, 7.1)	6.6 (6.3, 7.0)	6.8 (6.5, 7.3)	0.137
HbA1c(mmol/mol)	50 (46, 54)	49 (45, 53)	51 (48, 56)	0.137
UA (umol/L)	300.3 (249.8, 356.9)	321.2 (267.7, 374.7)	261.7 (220.1, 303.3)	<0.001
Cr (umol/L)	65.9 (55.7, 79.8)	75.1 (64.5, 87.7)	53.9 (46.9, 61.9)	<0.001
eGFR (mL/min)	71.4±16.8	70.0±16.2	73.4±17.3	0.152
uACR (mg/gCr)	15.5 (7.6, 45.1)	13.9 (6.8, 82.4)	16.7 (10.0, 37.1)	0.481
uL-FABPCR (μg/gCr)	2.52 (1.60, 4.65)	2.55 (1.47, 5.02)	2.76 (1.92, 4.61)	0.648
SAF (AU)	2.4 (2.1, 2.6)	2.4 (2.2, 2.7)	2.3 (2.1, 2.6)	0.158
Current smoking (n, (%))	40 (18.2)	37 (28.9)	3 (3.3)	<0.001
Hypertension (n, (%))	148 (67.3)	81 (63.3)	67 (72.8)	0.148
Dyslipidemia (n, (%))	159 (72.3)	88 (68.8)	71 (77.2)	0.222
Duration of T2D (years)	11 (6, 18)	11 (5, 18)	10 (6, 18)	0.601
ARB or ACEi (n, (%))	98 (44.5)	56 (43.8)	42 (45.7)	0.785
CCB (n, (%))	79 (35.9)	47 (36.7)	32 (34.8)	0.778
β blocker (n, (%))	5 (2.3)	3 (2.3)	2 (2.2)	0.999
MR blocker (n, (%))	1 (0.4)	0 (0)	1 (1.1)	0.418
Statin (n, (%))	118 (53.6)	59 (46.1)	59 (64.1)	0.009
Ezetimibe (n, (%))	18 (8.1)	8 (6.3)	10 (10.9)	0.225
Other hypolipidemic drugs (n, (%))	8 (3.6)	6 (4.7)	2 (2.2)	0.473

Antiplatelets (n, (%))	17 (7.7)	14 (10.9)	3 (3.3)	0.041
SU or Glinide (n, (%))	45 (20.5)	31 (24.2)	14 (15.2)	0.128
Metformin (n, (%))	139 (63.2)	77 (60.2)	62 (67.4)	0.322
DPP-4i (n, (%))	149 (67.7)	86 (67.2)	63 (68.5)	0.884
SGLT2i (n, (%))	102 (46.4)	57 (44.5)	45 (48.9)	0.584
αGI (n, (%))	34 (15.5)	21 (16.4)	13 (14.1)	0.708
Pioglitazone (n, (%))	7 (3.2)	4 (3.1)	3 (3.3)	0.999
Insulin (n, (%))	34 (15.5)	21 (16.4)	13 (14.1)	0.7080
GLP-1RA (n, (%))	18 (8.2)	11 (8.6)	7 (7.6)	0.999

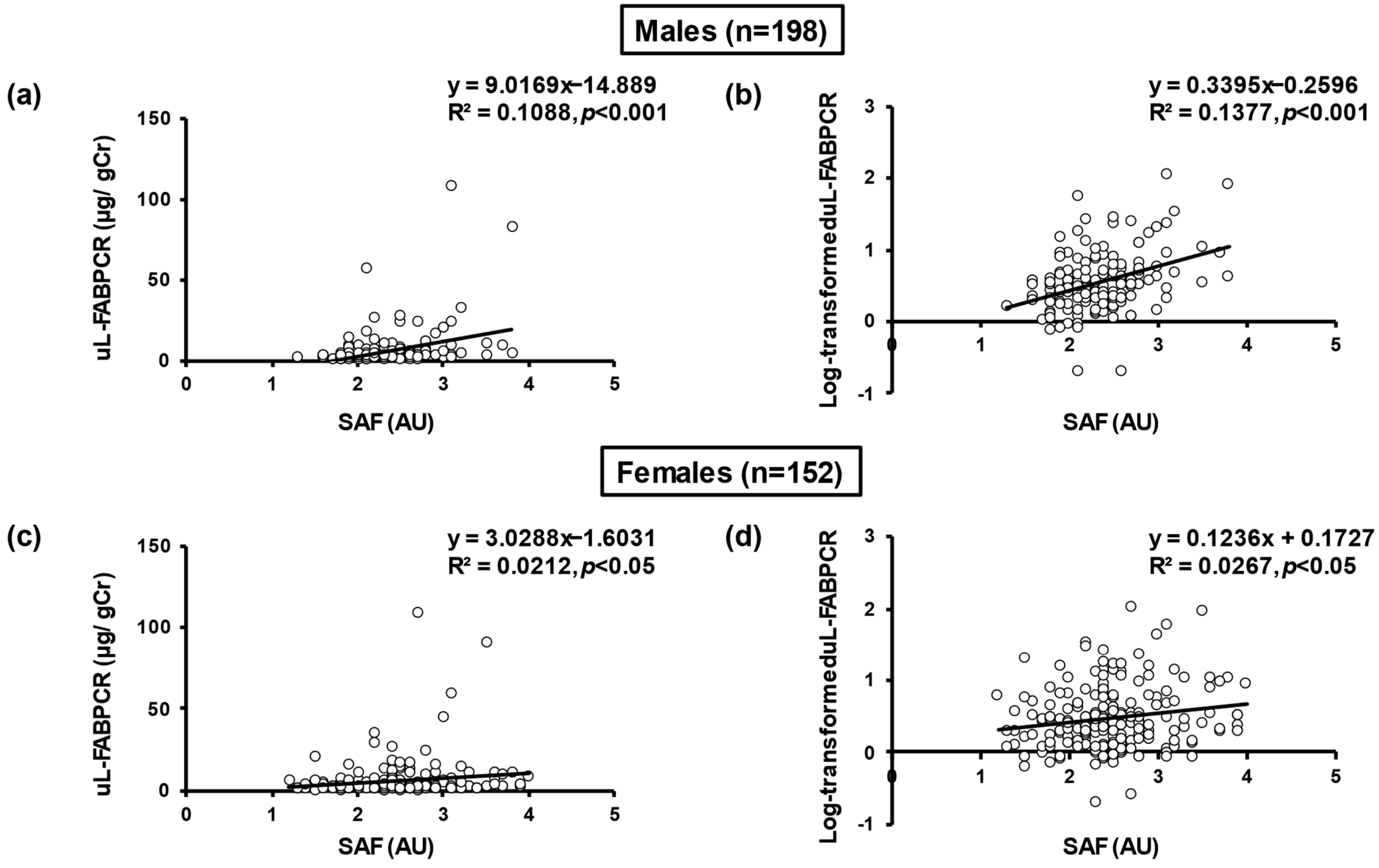

Figure S1. Associations of SAF value with uL-FABPCR and log-transformed uL-FABPCR in sex subgroup

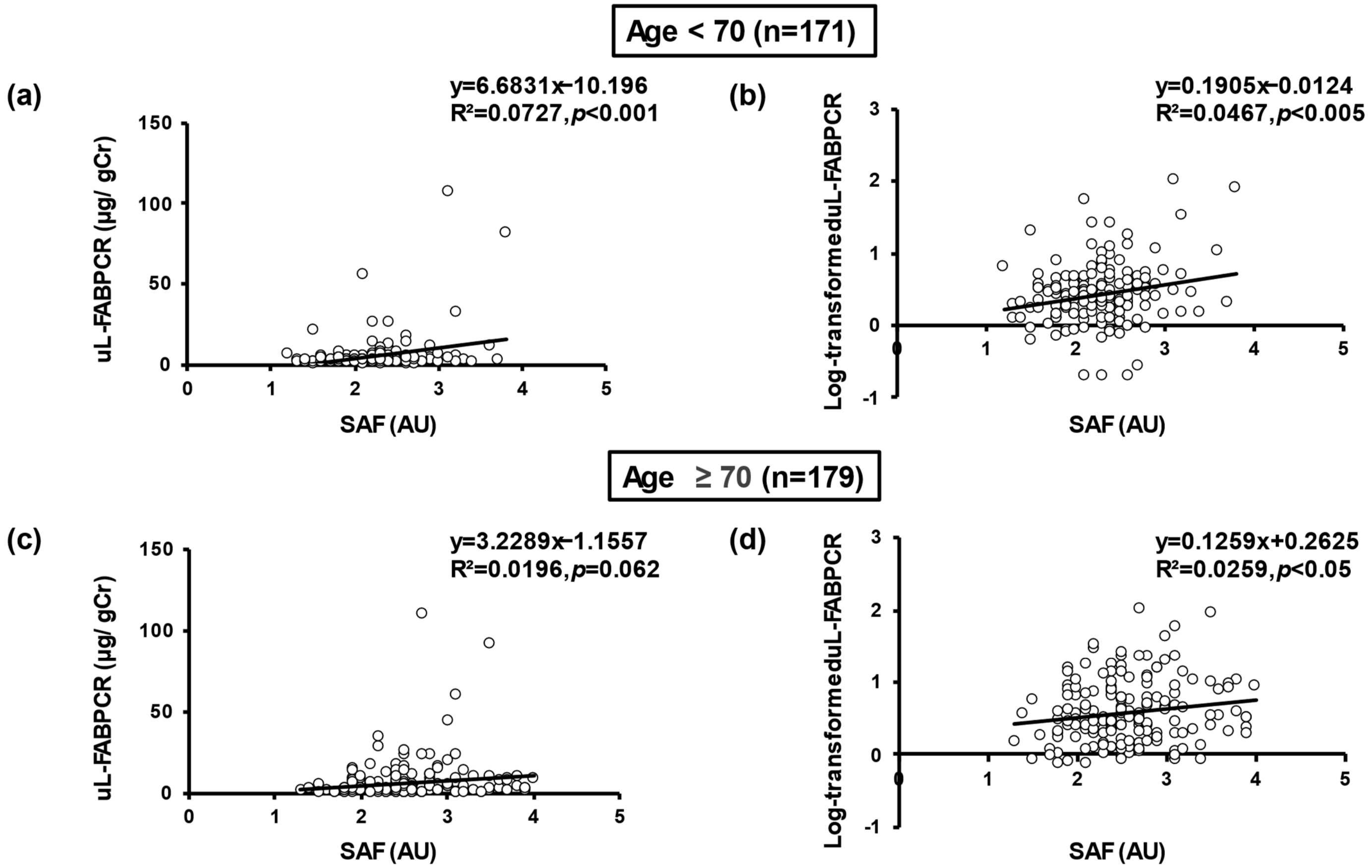

Figure S2. Associations of SAF value with uL-FABPCR and log-transformed uL-FABPCR in age subgroup

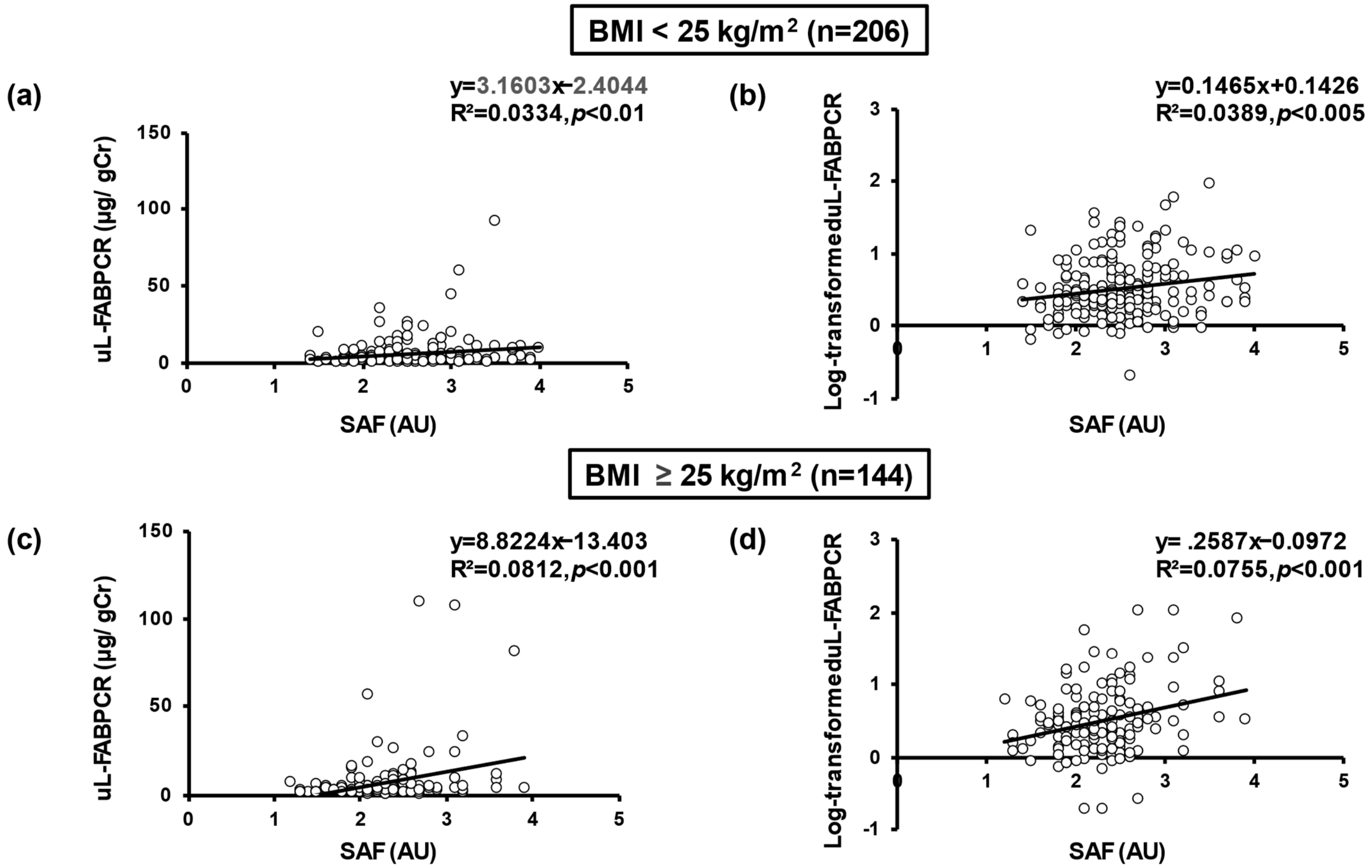

Figure S3. Associations of SAF value with uL-FABPCR and log-transformed uL-FABPCR in BMI subgroup

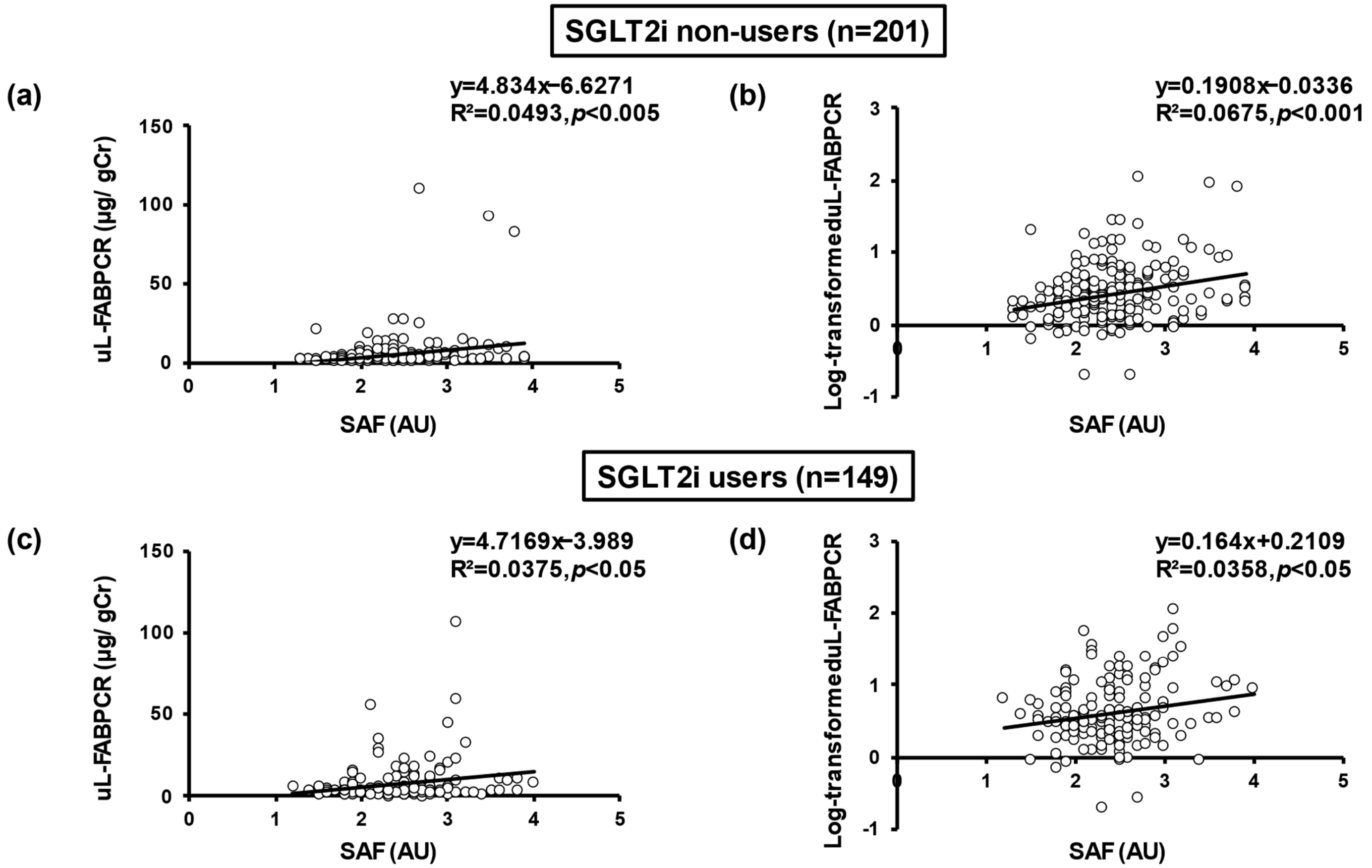

Figure S4. Associations of SAF value with uL-FABPCR and log-transformed uL-FABPCR in SGLT2i subgroup

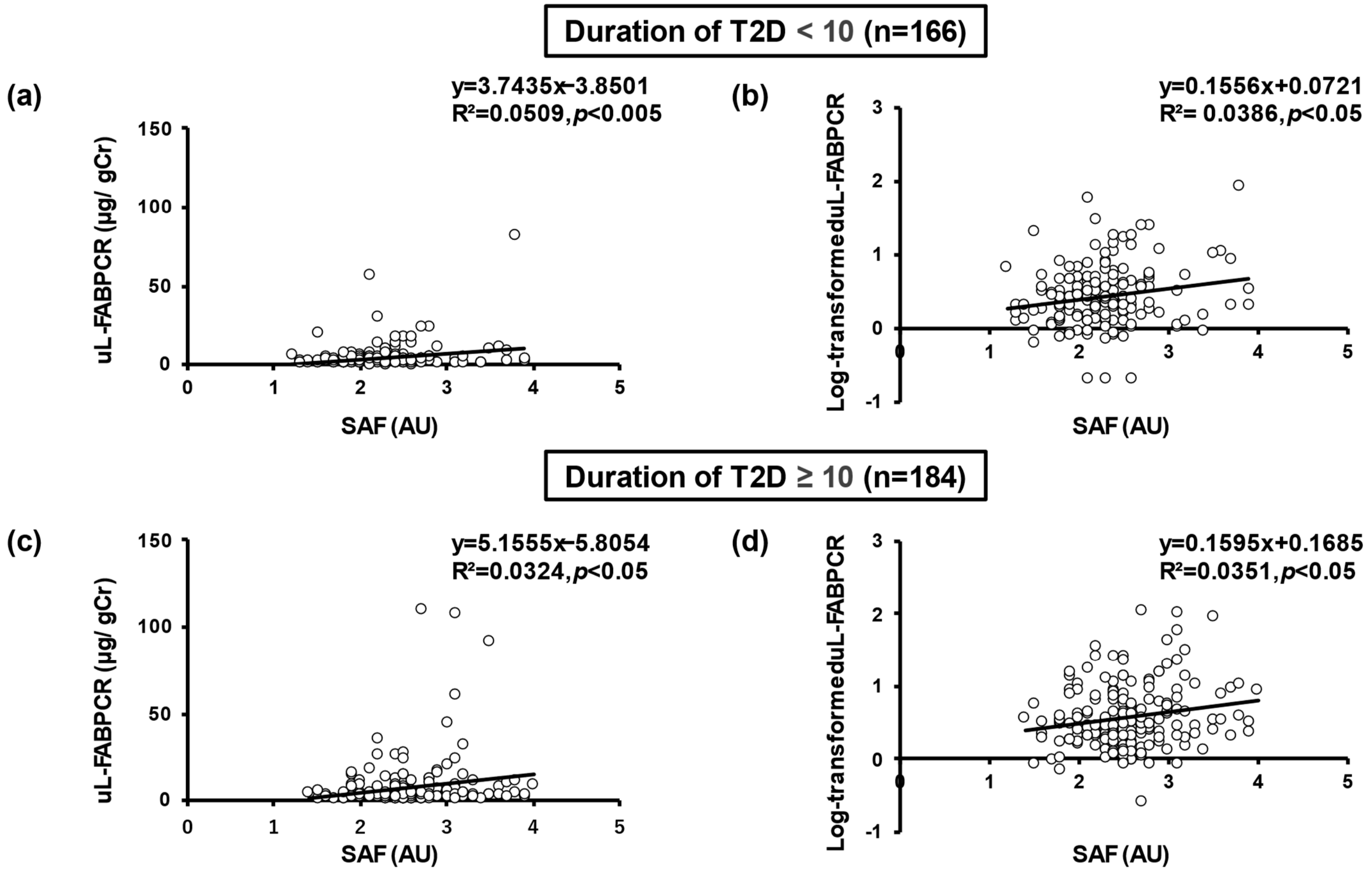

Figure S5. Associations of SAF value with uL-FABPCR and log-transformed uL-FABPCR in duration of T2D subgroup
